# Supplementary material for: The Systems Biology Research Tool: evolvable open-source software
Source: BMC Syst Biol. 2008 Jun 29;2:55. doi: 10.1186/1752-0509-2-55 (PMC2446383; doi:10.1186/1752-0509-2-55)
Supplement: Additional file 1 — SBRT Archive. An archive of the current version of the Systems Biology Research Tool. [file 1752-0509-2-55-S1.zip › sbrt-1.4.0/doc/developers_guide/api/sbrt/kernel/io/package-summary.html]

sbrt.kernel.io


|  |  |  |
| --- | --- | --- |
| **Overview** \| **Package** \| Class \| **Use** \| **Tree** \| **Deprecated** \| **Index** \| **Help** | |  |
| **PREV PACKAGE**   **NEXT PACKAGE** | **FRAMES**    **NO FRAMES**     **All Classes** |  |


---

## Package sbrt.kernel.io

Provides classes and interfaces used to perform kernel-level I/O.

**See:**
  
          **Description**

| **Interface Summary** | |
| --- | --- |
| **ResultsManager<T>** | This interace is used to represent a results-manager. |
| **WarningHandler** | This interface is used to represent a class of objects that deal with warning messages. |

| **Class Summary** | |
| --- | --- |
| **AbstractResultsManager<R>** | This class is a skeleton implemenation of `ResultsManager`. |
| **ListResultsManager<R>** | This class is used to store a list of results that can be easily retrieved. |
| **SingleRm<R>** | This class is used to store a single result that can be easily retrieved. |
| **WarningHandlerFactory** | This class is used to provide a default `WarningHandler`. |

| **Exception Summary** | |
| --- | --- |
| **ConflictingInputException** | This class is used to indicate conflicting inputs. |

## Package sbrt.kernel.io Description

Provides classes and interfaces used to perform kernel-level I/O.

---


|  |  |  |
| --- | --- | --- |
| **Overview** \| **Package** \| Class \| **Use** \| **Tree** \| **Deprecated** \| **Index** \| **Help** | |  |
| **PREV PACKAGE**   **NEXT PACKAGE** | **FRAMES**    **NO FRAMES**     **All Classes** |  |


---
